# Early Outcomes of Laparoscopic Transabdominal Preperitoneal (TAPP) Repair

**DOI:** 10.7759/cureus.35567

**Published:** 2023-02-28

**Authors:** Siddique Ahmad, Raashid Aslam, Muhammad Iftikhar, Muhammad Alam

**Affiliations:** 1 Department of General Surgery, Hayatabad Medical Complex Peshawar, Peshawar, PAK

**Keywords:** preperitoneal, recurrence, complication, lichtenstein, inguinal hernia, tapp

## Abstract

Background

Inguinal hernia repair is one of the commonest general surgical procedures performed all over the world. The introduction of synthetic mesh and laparoscopic repair has revolutionized inguinal hernia surgery in the recent past. Laparoscopic transabdominal preperitoneal (TAPP) repair is now considered a well-established procedure with minimal complications and short hospital stay and less recurrence. The TAPP approach gives a good view of the inguinal anatomy and a better understanding of the sac contents. The learning curve associated with TAPP repair is much less as compared to total extraperitoneal (TEP) repair. The aim of this study was to assess the effectiveness of TAPP repair for inguinal hernia in terms of the duration of surgery, hospital stay, complications, and recurrence rate.

Method

From March 1, 2019, to February 28, 2021, a total of 60 patients with inguinal hernias between ages 25 and 70 years were included in the study. A preoperative anesthesia assessment was done, and informed written consent was taken from all patients. The TAPP procedure was performed with polypropylene mesh in all cases, and surgery was performed by a surgeon with more than five years of laparoscopic experience.

Results

The total number of patients included in the study was 60. All patients were male. The mean±standard deviation (SD) age of the patients was 54.6±11.4 years. Unilateral primary inguinal hernia was present in 46 (76.6%) cases, recurrent in eight (13.3%) cases, and primary bilateral in six (10%) cases. The mean±SD duration of surgery for unilateral inguinal hernia was 59.1±15.7 minutes, and for bilateral hernia, it was 83.5±12.6 minutes. The mean hospital stay was 3.6±1.5 days. Scrotal swelling was noted as a common complication in seven (11.6%) cases, surgical site infection (SSI) in three (5%), mesh infection in two (3.3%), urinary retention in two (3.3%), and chronic pain in one (1.6%). No recurrence was noted.

Conclusion

Transabdominal preperitoneal repair for inguinal hernia is a very effective procedure with a short learning curve and minimal complication rate. The hospital stay is less, and recurrence is very low.

## Introduction

Inguinal hernia repair is one of the most common surgical procedures performed worldwide. It has transformed from tissue repair to Darning, to mesh repair over the years. Lichtenstein mesh repair was considered the gold standard technique until recently. With the introduction and popularity of laparoscopes, surgeon and patient preferences have changed toward laparoscopic repair of hernia very rapidly [1].

The laparoscopic approach for inguinal hernia was started in the 1990s and was adopted rapidly by surgeons all over the world. There are different laparoscopic approaches to the inguinal hernia, i.e., transabdominal preperitoneal (TAPP), total extraperitoneal (TEP), and intraperitoneal onlay mesh (IPOM). With the passage of time, IPOM has lost its popularity as a procedure of choice for inguinal hernia repair, but TAPP and TEP have proved to be effective day by day [2]. Laparoscopic surgery has many advantages over open surgery, but a steep learning curve and prolonged duration of surgery are the two common disadvantages of the laparoscopic approach [3].

TAPP repair is considered a well-standardized procedure for the treatment of inguinal hernia and is especially effective in the case of bilateral hernia where a patient is prevented from two groin incisions of the open repair. Because of minimal tissue trauma, recovery is rapid, and return to work is quick. TAPP repair is a good alternative in recurrent cases where the previous repair was done through the anterior approach as the preperitoneal space is spared and dissection will be easy in this area [4]. It also gives the advantage of identifying any concurrent femoral hernia, contralateral hernia, or even obturator hernia. The use of wide mesh in the preperitoneal space covers other possible hernia sites such as femoral hernia and obturator hernia. Adhering to the principles of minimally invasive surgery, following a critical view of the myopectineal orifice in the dissection and use of large-sized mesh, has largely eliminated complications in TAPP repair [5].

The purpose of this study is to ascertain the effectiveness of TAPP and share our experience with this procedure in terms of the duration of surgery, hospital stay, complications, and recurrence rate.

## Materials and methods

This prospective study was conducted in the Surgical Department of Hayatabad Medical Complex Peshawar from March 1, 2019, to February 28, 2021. After taking approval from the Hospital Ethical Committee under reference number 1182, all patients who presented to the Outpatient Department (OPD) with inguinal hernia, whether primary, recurrent, unilateral, or bilateral, from the 25 to 70 years age group, were included in the study. Patients with obstructed/strangulated hernia and very large inguinoscrotal hernia and patients unfit for anesthesia with American Society of Anesthesiologists (ASA) physical status IV were excluded from the study. Informed written consent was taken from all patients before surgery, and anesthesia assessment was done. General anesthesia was used in all patients, and surgery was performed by a surgeon with more than five years of experience in both open and laparoscopic surgery. The follow-up period was one year.

Surgical technique

**Figure 1:**
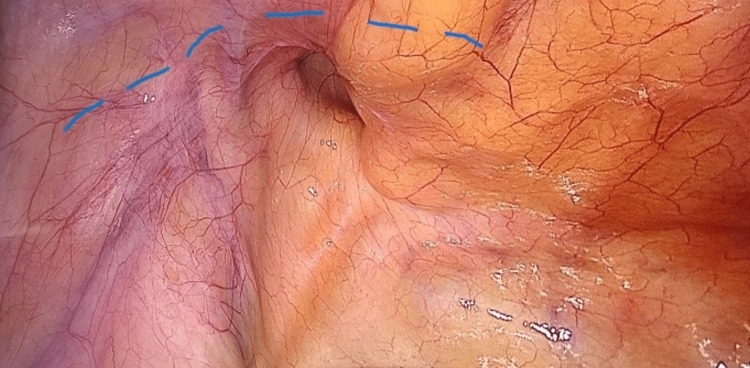
Peritoneal incision line

**Figure 2:**
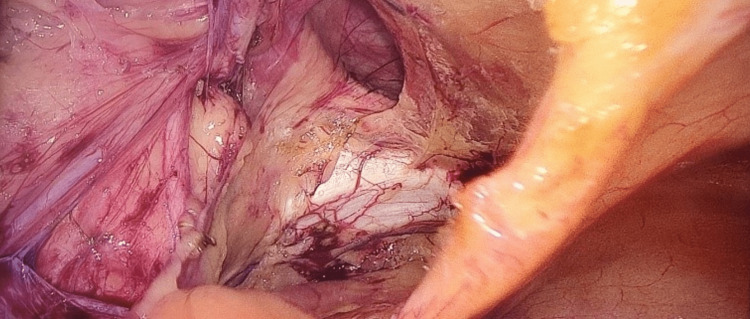
Dissection of the myopectineal orifice

**Figure 3:**
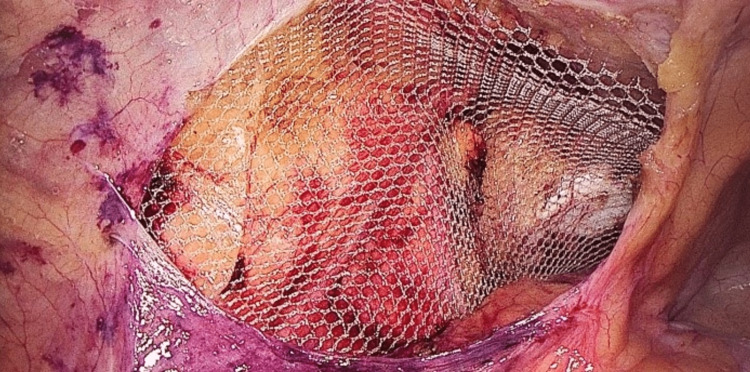
Mesh in the preperitoneal space

**Figure 4:**
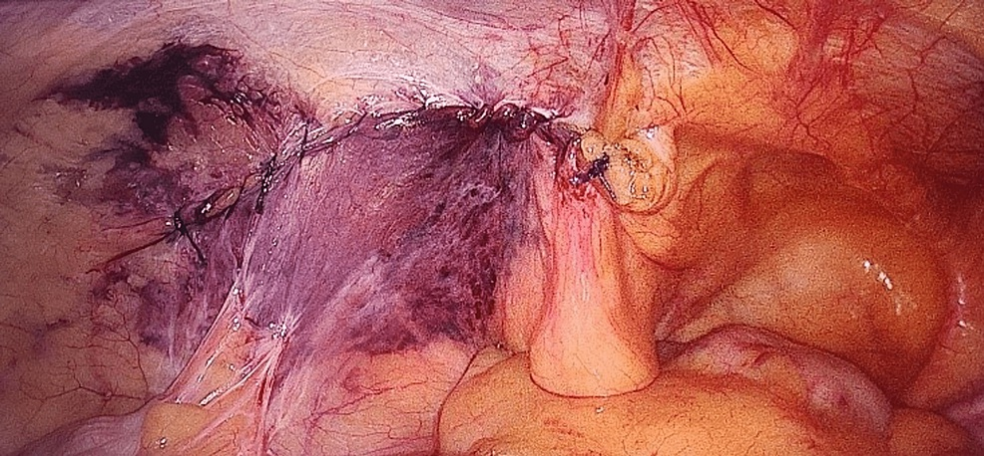
Peritoneal flap closure

The procedure was performed using three ports. The first port (10 mm) is placed above the umbilicus and used for creating pneumoperitoneum. Then, a telescope is entered, and the peritoneal cavity is inspected; the hernia is then confirmed. Two 5-mm ports were then placed on either side lateral to the rectus sheath. The ipsilateral port is in line with the optical port, while the contralateral port is 2 cm below. A peritoneal incision was performed using an endoscopic scissor attached with monopolar cautery 3-4 cm above the deep ring (Figure 1). The sac is dissected, and the preperitoneal space is created (Figure 2). Polypropylene mesh (10×15) is placed in the preperitoneal space and fixed with vicryl 2/0 at two points (Figure 3). The peritoneal flap was closed with vicryl 2/0 continuous sutures (Figure 4). The skin is closed with prolene 2/0. The duration of the surgery was noted in minutes from the first incision to skin closure.

## Results

A total of 60 patients, all male, were prospectively recruited to undergo TAPP repair. The mean age was 54.6±11.4 years. There were 29 (48.3%) right-side primary hernias, 17 (28.3%) left side, and six (10%) bilateral. There were a total of eight (13.3%) patients with recurrent hernias, five on the right side and three on the left. There were 37 (61.6%) direct hernias and 23 (38.3%) indirect hernias (Table 1). The duration of surgery for unilateral inguinal hernia was 59.1±15.7 minutes, and for bilateral hernia, it was 83.5±12.6 minutes. The mean postoperative hospital stay was 3.6±1.5 days. Postoperative complications include scrotal swelling in seven (11.6%) cases, urinary retention in two (3.3%), surgical site infection (SSI) in three (5%), mesh infection in two (3.3%), and chronic pain in one (1.6%). No recurrence was noted in this study, and mortality was nil (Table 2).

**Table 1:** Patient characteristics

Patient characteristic	Frequency (%)
Male	60 (100%)
Female	0 (0%)
Primary hernia (right)	29 (48.3%)
Primary hernia (left)	17 (28.3%)
Recurrent (right)	5 (8.3%)
Recurrent (left)	3 (5%)
Bilateral	6 (10%)
Direct inguinal hernia	37 (61.6%)
Indirect inguinal hernia	23 (38.3%)

**Table 2:** Postoperative complications

Complication	Frequency
Scrotal swelling	7 (11.6%)
Urinary retention	2 (3.3%)
Surgical site infection	3 (5%)
Mesh infection	2 (3.3%)
Chronic pain	1 (1.6%)
Hernia recurrence	0 (0%)
Total	15 (25%)

## Discussion

The last two decades have seen the introduction of different laparoscopic techniques in inguinal hernia repair. This has increased the interest of surgeons in inguinal hernia surgery. Although open Lichtenstein mesh repair has been considered very effective in the management of inguinal hernias, the optimal surgical approach remains controversial [6]. With the introduction of minimally invasive techniques, surgeons began adopting laparoscopic surgery in hernia management. IPOM, TEP repair, and TAPP repair are the three procedures used for the last many years. TEP repair and TAPP repair have become more popular in inguinal hernia repair. TEP repair is a totally extraperitoneal approach and has a long learning curve and cannot be done in every case [7]. TAPP repair is an abdominal approach with a clear view of the anatomy and the advantage of more space in the abdominal cavity. Because of this reason, TAPP repair is being practiced more frequently than TEP repair, especially by beginners [8].

The mean age of the patients in this study group was 54.6±11.4 years (range: 42-68 years). This result shows the general concept that the occurrence of inguinal hernia increases with age as predisposing factors for hernia are more in the elderly, such as chronic cough, constipation, and benign prostatic hyperplasia (BPH) [9]. The results are similar to other studies, such as those of Thanh Xuan and Huu Son [10], where the average age was 60.4±11.85 years, and Peitsch, with an average age of 59.1 years [11]. Right-side inguinal hernias were more common (29 (48.3%)) than left (17 (28.3%)) and bilateral (6 (10%)) hernias, which corresponds to other studies [10]. Out of a total of 60 patients, 52 (86.6%) had a primary inguinal hernia, and eight (13.3%) had a recurrent hernia. In the recurrent cases, five had been operated on with the Lichtenstein method and three with modified Bassini’s repair (Darning with prolene suture). We came across no recurrence after TAPP repair in our study. The reason may be that TAPP repair is relatively new in our area and not many surgeons routinely practice TAPP repair here. While doing TAPP repair in recurrent cases, it was particularly noted that there was no difficulty in dissection and placing mesh in the preperitoneal space because the anatomical structures were intact as in primary cases [12]. The mean hospital stay was 3.6±1.5 days. Hospital stay is somewhat higher in this study because patients who undergo general anesthesia are admitted a day before surgery as per hospital policy. Even then, it is significantly lower as compared to open Lichtenstein mesh repair because of minimal tissue trauma [13].

The mean time of procedure (TAPP repair) for unilateral inguinal hernia was 59.1±15.7 minutes, and that for bilateral hernia was 83.5±12.6 minutes. This is more or less the same as in other studies [10,11]. As TAPP repair is associated with a steeper learning curve as compared to open, the duration of surgery is obviously high initially; however, with experience and a better understanding of the inguinal anatomy, it can be reduced significantly. One of the advantages of TAPP repair noted in this study was that we identified two cases of contralateral subclinical hernias that were treated simultaneously. We used a lightweight prolene mesh measuring 10×15 cm in all patients. It is recommended to use large-sized mesh to prevent recurrence and other hernias such as femoral and obturator hernias [13].

The early complications noted in the study were urinary retention in two (3.3%) cases and SSI in three (5%) cases, and all were related to the supra-umbilical optical port. Scrotal swelling was also observed in seven (11.6%) cases and mesh infection in two (3.3%) cases. One of the patients presented after one year with a huge collection in the area of mesh with groin pain and no fever. After a CT scan, he was treated with percutaneous aspiration by a radiologist, and the pus yielded no growth on culture. He did not require taking out the mesh. The second case of mesh infection presented early within two weeks with fever and leukocytosis. He was treated with incision drainage and mesh removal using the anterior approach along with broad-spectrum antibiotics according to the culture report. At six-month follow-up, one patient reported pain in the groin of moderate intensity, and no recurrence was noted after one-year follow-up [14].

## Conclusions

Laparoscopic TAPP repair of inguinal hernia is a safe and effective procedure. The use of prolene mesh in the preperitoneal space also has the additional advantage of preventing the future development of femoral and obturator hernia. TAPP repair is particularly good in bilateral cases and in recurrent cases after failed anterior repair. The operative time, complications, and recurrence is related to the experience of the surgeon.
